# Intensive rehabilitation after pelvic and hip fractures: a comparative retrospective study

**DOI:** 10.3389/fmed.2024.1346354

**Published:** 2024-05-16

**Authors:** Dori Katz, Alex Geftler, Ahmed Abu-Ajaj, Evgeni Makulin, Eva Star, Evgeniya Zikrin, David Shacham, Natalia Velikiy, Tamar Freud, Yan Press

**Affiliations:** ^1^Faculty of Health Sciences, Ben-Gurion University of the Negev, Be'er Sheva, Israel; ^2^Department of Geriatrics, Soroka Medical Center, Be'er Sheva, Israel; ^3^Department of Orthopedics, Soroka Medical Center, Be'er Sheva, Israel; ^4^Department of Family Medicine, Faculty of Health Sciences, Ben-Gurion University of the Negev, Be'er Sheva, Israel; ^5^Siaal Research Center for Family Medicine and Primary Care, Faculty of Health Sciences, Ben-Gurion University of the Negev, Be'er Sheva, Israel; ^6^Unit for Community Geriatrics, Division of Health in the Community, Ben-Gurion University of the Negev, Be'er Sheva, Israel; ^7^Center for Multidisciplinary Research in Aging, Ben-Gurion University of the Negev, Be'er Sheva, Israel

**Keywords:** pelvic fracture, hip fracture, predictors, intensive rehabilitation, inpatients

## Abstract

**Purpose:**

Pelvic fracture (PF) is common, especially among older patients, and its prevalence increases over time. In contrast to hip fracture (HF), the literature on rehabilitation after PF is scant, mandating a study of the outcomes of rehabilitation in patients with PF. The present study compared patients who underwent intensive rehabilitation following HF or PF.

**Methods:**

A retrospective study of patients 65 years of age and older who underwent intensive rehabilitation in the Geriatrics Department. Data were collected on patients with PF, while data on patients with HF were taken from an earlier study. All patients in both groups suffered from low-energy trauma. Rehabilitation outcomes were measured using the Montebello Rehabilitation Factor Score-revised (MRSF-R).

**Results:**

144 PF patients were compared with 138 HF patients. The mean age of the patients in the HF group was 82.5 ± 7.1 compared to 81.5 ± 6.9 in the PF group (*p* = 0.230). Females comprised 77.5% of the patients in the HF group and 90.3% in the PF group (*p* = 0.04). All patients in the HF group underwent surgical repair of their fracture, while all patients in the PF group had non-surgical treatment. More patients in the HF sample had a nursing caregiver prior to the fracture (92.0% vs. 60.4%, *p* < 0.001), had a higher Charlson Co-morbidity Index total score (2.1 ± 1.9 vs. 1.6 ± 1.7, *p* = 0.13), developed more delirium (21.7% vs. 8.3%, *p* = 0.16), more infections (29.0% vs. 11.1%, *p* < 0.002), and more cardiovascular complications (23.9% vs. 5.6%, *p* < 0.001) during hospitalization. They had longer hospital stays (20.9 ± 7.5 vs. 18.2 ± 7.7 days, *p* = 0.0007), and had a higher mortality rate (13.8% vs. 6.3%, *p* = 0.037) over the first year following the fracture than the PF group. A similar rate of patients in both groups (64.5% vs. 60.4%, *p* = 0.483) had successful intensive rehabilitation. In the PF group only cognitive state was an independent predictor of successful rehabilitation, with each additional point in the Mini Mental State Examination (MMSE) increasing the patient’s chance of reaching an MRFS-R score ≥ 50 by 20.5%.

**Conclusion:**

Despite slightly different characteristics in the two groups, the results of intensive rehabilitation were similar. Cognitive state was the only independent factor that affected achievement of a better rehabilitation outcome. With the increasing rate of PF, more studies should focus on rehabilitation in this patient population.

## Introduction

Hip fracture (HF) is a very common problem throughout the world (1). In elderly individuals it often leads to deterioration in their general condition (2–4). The risk of death in the first year after HF is threefold that of the same age group without HF (5). Many patients experience a decline in physical, social, and mental function and only half of the patients succeed in regaining their pre-HF functional capacity (6, 7). The recuperation period often is accompanied by emotional and social crises, in some cases leading to suicidal ideation (8).

Pelvic fractures (PF) are usually associated with high-energy trauma, are complex, difficult to treat and, at times, have a poor prognosis. They are considered the major cause of mortality among types of skeletal traumas (9). PF are also a common problem in the adult population (10–14).

The rate of PF among all low-energy fractures patients has increased over the years (10, 11, 13). While the rate of HF is on the decline, there is an opposite trend for PF. A nationwide register study from Sweden showed a decline of 18% in the incidence of HF and an increase of 25% in the incidence of PF over the last 16 years (15).

A large proportion of PF patients (11–53%) develop complications associated with PF over the course of hospitalization or after discharge (10, 15–21), with limited mobility (9, 12, 22–25), increased long-term care (LTC) (10, 12, 17, 19, 23, 24), decreased quality of life (16), increased utilization of healthcare system resources (14), and a substantial increase in mortality (9–12, 14–18, 20–24, 26–29).

Compared to the literature on HF, the literature on PF is scant even though the outcomes of the two types of fractures, for example LTC and mortality, are similar (30). Only a few studies, investigated predictors of mortality following PF: age (11, 16, 27, 31), male sex (10, 11, 21), pre-fracture mobility (18, 21), co-morbidity (10, 12, 27), and type or number of PFs are predictors of death (10, 16).

The literature on rehabilitation outcome following HF is copious. In contrast, the literature on rehabilitation following PF is scant. Keene et al. (32) conducted a systematic review to evaluate clinical trials in patients with a fragility fracture of the pelvis or lower limb. They identified 37 studies, none of which was related to PF. Marrinan et al. (25) followed 110 patients who were hospitalized with PF and found that older age and lengthy hospital stay were independent predictors that a patient would need LTC at discharge from the hospital. Pfeiffer et al. (33) investigated the effect on rehabilitation outcomes of interventions aimed at reducing the fear of falling and increasing physical activity. Only 17 of the 115 study participants had PF. Sherrington et al. (34) evaluated the effect of exercise on limited motility and fall prevention in patients with fractures in their lower limbs and PF. They did not assess or report on other variables that could affect the outcome of rehabilitation. Only 31 of 336 study patients had PF and only 15 of the 336 began intervention less than 3 months after the fracture. To our knowledge, no studies have been published that specifically assessed the results of rehabilitation after PF. The present study had two main objectives: (a) to characterize patients who underwent intensive rehabilitation following PF and to compare them to patients who underwent the same treatment following HF and, (b) to identify predictors of successful rehabilitation following PF.

## Methods

### Setting and study population

The study was conducted in the Geriatrics Department of the Soroka University Medical Center (SMC), a tertiary care center in southern Israel. The department has 25 hospital beds with a multidisciplinary staff including board certified specialists in geriatric medicine, doctors training in geriatric medicine, nurses, physical therapists, occupational therapists, a social worker, and nutritionists. The department’s activities focus, in part, on intensive rehabilitation for patients 65 years of age and above who suffered a fracture.

Patients with HF are admitted from orthopedic surgery departments in the first days after the fracture. The process of admission to these departments has been described in the past (35). Briefly, these patients are assessed initially while hospitalized in the orthopedic surgery departments in the first days following surgery and patients who meet the requirements for rehabilitation in the Geriatrics Department are transferred to it. Patients after PF who do not require surgical intervention and meet the criteria for rehabilitation in our department are admitted directly from the emergency room on the basis of available beds. If there are no available beds they are admitted to another ward, usually orthopedic surgery, from where they are transferred, when possible, to our department.

For patients in both HP and PF groups, there are consistent criteria for admission to rehabilitation in the Geriatric Department: they are not able to undergo rehabilitation in the community, there are multiple geriatric symptoms, their health maintenance organization (HMO) covers rehabilitation in the department, and the patients agree to undergo the program in the department.

It should be emphasized that no patients with PF in this study underwent any type of surgical intervention. In contrast, all patients in the HF group had undergone surgical repair of their fractures before being admitted to the Geriatrics Department.

While in the Geriatrics Department, PF patients, who did not have surgery, and HF patients, who underwent surgical repair, went through an identical process including a comprehensive geriatric assessment by the multidisciplinary staff, which is the basis for the rehabilitation plan and treatment, and weekly staff meetings in which the patients’ progress and suitability for the program were reviewed. The rehabilitation process included mobilization facilitated by all staff members, physiotherapy five times per week (average 45 min per session), occupational therapy several times a week (average 30 min per session), and a psychosocial intervention by the social worker. The end of intensive treatment and discharge from the ward were based on the patient’s ability to make transfers, to be mobile, and to use the toilet without help, or when the patient’s rehabilitation graph reached a plateau level.

At the completion of the intensive rehabilitation program in the department, the staff arranged for rehabilitation treatment in the community, or transfer of the patient to an LTC framework.

Men and women, 65 years of age and above, who were hospitalized for rehabilitation in the Geriatrics Department following PF between Jan. 1, 2012 and Aug. 31, 2020 were included in the study. For comparison we used a sample population that was included in our previous study (35), in which we collected data on patients between Jan. 1, 2016 and Dec. 31, 2019. All patients in the HF and PF groups had fall-induced fractures, i.e., low-energy trauma.

### Data collection methods

A retrospective survey of the computerized medical records of the patients was conducted and the following data were collected:

Demographic variables: age, sex, education, family status, place of residence, and the amount of welfare allocation prior to the event.

### Clinical variables

- In the case of PF, the type of fracture by the AO/OTA classification (36).
- Co-morbidity. This was calculated using the Charlson’s Comorbidity Score (CCI) (37) including the total score (TS) and the total cumulative score (TCS), which was the sum of the TS and the patient’s age.
- The number of medications that the patient was taking prior to admission to the Geriatrics Department.
- The patient’s Body Mass Index (BMI) at admission to the Geriatrics Department.
- The Norton index (38) to calculate the risk of developing a pressure sore.
- Lab tests results on admission to the Geriatrics Department: hemoglobin, serum albumin, creatinine and urea.
- Cognitive assessment using the Mini Mental State Examination (MMSE) (39).
- Depression was diagnosed by the staff doctors using the Diagnostic and Statistical Manual of Mental Disorders 5th edition (DSM-5) criteria (40).
- Complications that developed over the course of hospitalization: rate of delirium, infection, cardiovascular problems, thromboembolism, and pressure sores.
- Functional assessment was conducted using the Functional Independence Measure (FIM) (41), which was measured for anamnestic FIM (anFIM) that relates to the pre-fracture period, FIM on admission (FIMa), and FIM at discharge (FIMd).

### Data on hospitalization and mortality

The length of hospitalization in the Geriatrics Department.

Mortality over the first post-fracture year.

### Statistical analysis

Statistical analyses were conducted with the IBM SPSS Statistical Program, version 25. The results are presented as mean ± standard deviation for continuous variables with a normal distribution, as median and the interquartile range for continuous variables with a non-normal distribution, and as percentages for qualitative variables. We calculated the Montebello Rehabilitation Factor Score-Revised (MRFS-R) (42). This index is based on differences among anFIM, FIMa, and FIMd, using the following formula:


MRFS−R=((FIMd−FIMa)/(FIMd))/((anFIM−FIMa)/(anFIM)).

Successful rehabilitation was defined as great than or equal to 50%.

A univariate analysis was performed to evaluate associations between demographic and clinical variables and the outcome of rehabilitation. Student’s *t*-test or One-way ANOVA were used for continuous variables with normal distribution. If the distribution was not normal Mann–Whitney or Kruskal-Wallis were used for these variables. The Chi-Square test was used for qualitative variables.

Variables with a significant difference in univariate analyses (*p* < 0.1) and those with clinical significance were included in a multivariate model. Before this analysis was done, a check was conducted for interactions or confounders, which were removed from the model, when deemed necessary.

The study was approved by the Internal Review Board (Helsinki Committee) of the Soroka University Medical Center (Approval # SOR-0466-20; date of registration: 18/05/2021).

## Results

The study population included 144 PF patients and 138 HF patients. As stated above, the HF group has been described in the past (35), and is presented here only as a comparison group for the PF sample. In short, the mean age of the HF patients was 82.5 ± 7.1 years and 107 (77.5%) were women. Forty two (30.4%) had intracapsular fractures and 96 (69.6%) had other types of extracapsular HF. Ninety five (68.8%) underwent internal fixation, 21 (15.2%) total hip replacement, and 22 (15.9%) hemiarthroplasty.

Table 1 summarizes data for the two groups. The mean age of PF patients was 81.5 ± 6.9 compared to 82.5 ± 7.1 for the HF group (*p* = 0.23). The percentage of women was significantly higher in th PF group (90.3% vs. 77.5%, *p* < 0.001) and significantly fewer in that group required nursing care prior to the fracture (60.4% vs. 92.0%, *p* < 0.001). The HF group had significantly higher co-morbidity levels than the PF group based on the Charlson Co-morbidity Index, total score (CCI TS) (2.1 ± 1.9 vs. 1.6 ± 1.7, *p* = 0.013) and the Charlson Co-morbidity Index, total cumulative score (CCI TCS) (5.9 ± 1.9 vs. 5.3 ± 1.8, *p* = 0.003). Patients in the HF group were taking significantly more drugs prior to admission to the department than PF patients (10.2 ± 3.3 vs. 6.8 ± 3.7, *p* < 0.0001). Compared to the PF group, patients in the HF group were at a significantly higher risk to develop pressure sores based on the Norton index (14.1 ± 1.3 vs. 13.6 ± 1.5, *p* = 0.003).

**Table 1:** Socio-demographic and medical characteristics.

	Hip fracture (*N* = 138)	Pelvic fracture (*N* = 144)	*p*
**Age (years)** (Mean ± SD)	82.5 ± 7.1	81.5 ± 6.9	0.230
**Gender (female)** [*n* (%)]	107 (77.5)	130 (90.3)	0.004
**Family status (married)** [*n* (%)]	46 (33.3)	41 (28.5)	0.381
**Nursing caregiver (yes)** [*n* (%)]	127 (92.0)	87 (60.4)	<0.001
**Education > 12 years** [*n* (%)]	47 (34.1)	40 (27.8)	0.311
*Medical status*			
CCI TS (Mean ± SD)	2.1 ± 1.9	1.6 ± 1.7	0.013
CCI TCS (Mean ± SD)	5.9 ± 1.9	5.3 ± 1.8	0.003
Medication number (Mean ± SD)	10.2 ± 3.3	6.8 ± 3.7	<0.001
MMSE (Mean ± SD)	21.5 ± 6.3	22.3 ± 5.4	0.419
Depression [n (%)]	23 (16.7)	23 (16.0)	0.879
Norton (Mean ± SD)	13.6 ± 1.5	14.1 ± 1.3	0.003
BMI, (Mean ± SD)	24.7 ± 5.2	24.6 ± 4.6	0.910
*Laboratory*			
Hemoglobin (g/dl) (Mean ± SD)	12.1 ± 2.0	11.7 ± 1.7	0.100
Serum creatinine (mg/dl) (Mean ± SD)	1.4 ± 1.5	1.1 ± 0.9	<0.001
Serum urea (mg/dl) (Mean ± SD)	72.9 ± 37.8	54.5 ± 33.3	<0.001
Serum albumin (g/dl) (Mean ± SD)	3.0 ± 0.4	3.6 ± 0.5	<0.001
**Morbidity during rehab process** [all: n (%)]			
Delirium	30 (21.7)	12 (8.3)	0.016
Any infection	40 (29.0)	16 (11.1)	<0.002
Cardiovascular (IHD, CHF, stroke)	33 (23.9)	8 (5.6)	<0.001
Thromboembolism	3 (2.2)	1 (0.7)	0.351
Pressure Ulcers	4 (2.9)	3 (2.1)	0.683

CCI TS, Charlson Co-morbidity Index, total score; CCI TCS, the Charlson Co-morbidity Index, total cumulative score; MMSE, the Mini Mental State Examination; BMI, body mass index; IHD - ischemic heart disease; CHF, congestive heart failure.

Compared to the HF group, patients in the PF group had significantly lower creatinine levels (1.1 ± 0.9 vs. 1.4 ± 1.5, *p* = 0.0003), lower blood urea levels (54.5 ± 33.3 vs. 72.9 ± 37.8, *p* < 0.0001), and higher albumin levels (3.6 ± 0.5 vs. 3.0 ± 0.4, *p* < 0.0001).

The complications rate was significantly higher in the HF group. The most prevalent complications were delirium (21.7% vs. 8.3%, *p* = 0.0016), infection (29.0% vs. 11.1%, *p* = 0.0002), and cardiovascular (23.9% vs. 5.6%, *p* < 0.0001).

There were no statistically significant differences between the groups in the other socio-demographic, clinical (MMSE, depression rate or BMI), or laboratory variables. Data on functional capacity, the length of hospitalization in the Geriatrics Department, the rehabilitation outcome, and mortality are presented in Table 2.

**Table 2:** Functional status, rehabilitation outcome, hospitalization time, and mortality rate.

	Hip fracture (*N* = 138)	Pelvic fracture (*N* = 144)	*p*
*Functional status*			
anFIM (Mean ± SD)	111.3 ± 15.0	106.9 ± 18.2	0.090
FIMa (Mean ± SD)	65.1 ± 14.1	67.2 ± 15.7	0.243
FIMd (Mean ± SD)	83.6 ± 19.0	84.6 ± 18.09	0.756
**MRFS-R** (Mean ± SD)	47.9 ± 38.4	50.3 ± 43.6	0.414
Patients with MRFS-R ≥ 50 [*n* (%)]	89 (64.5)	87 (60.4)	0.483
**Length of stay in Geriatric Department (days)** (Mean ± SD)	20.9 ± 7.5	18.2 ± 7.7	0.0007
**Place of Discharge, LTC [*n* (%)]**	5 (3.6)	5 (3.5)	0.999
**1 year mortality** [n (%)]	19 (13.8)	9 (6.3)	0.037

anFIM, anamnestic Functional Independence Measure; FIMa, Functional Independence Measure on admission; FIMd, Functional Independence Measure on discharge; MRFS-R, the Montebello Rehabilitation Factor Score -Revised; LTC, Long Term Care.

There were no differences between the groups in anFIM, FIMa, and FIMd. The mean duration of rehabilitation was significantly longer in the HF group (20.9 ± 7.5 vs. 18.2 ± 7.7 days, *p* = 0.0007). The outcome of rehabilitation was similar in the two groups with 87 of 144 patients in the PF group (60.4%) and 89 of 138 in the HF group (64.5%) reaching an MRFS-R score ≥ 50 (*p* = 0.483).

The vast majority of the patients were discharged to their homes at the end of the rehabilitation process in the Geriatrics Department. Only five of 138 patients with HF (3.6%) and five (3.5%) of PF patients were transferred from the Geriatric Department to LTC (*p* = 0.999).

There was a statistically significant difference between the groups in one-year mortality with 19 deaths (13.8%) in the HF group compared to 9 (6.3%) in the PF group (*p* = 0.037).

Table 3 shows a comparison between PF patients with a successful rehabilitation and those whose rehabilitation was unsuccessful. There was a borderline difference in age with a mean age of 80.6 ± 7.4 in the successful group versus 82.9 ± 6.0 in the others (*p* = 0.055). The mean MMSE score was significantly higher in the successful group (24.2 ± 4.5 vs. 19.2 ± 5.3, *p* < 0.0001) and the rate of infections in the Geriatrics Department was significantly lower (5.7% vs. 19.3%, *p* = 0.015) in that group. There were no significant differences between the groups in the other variables.

**Table 3:** Predictors of MRFS-R ≥ 50 among PF patients.

	MRFS-R < 50 (*N* = 57)	MRFS-R ≥ 50 (*N* = 87)	*p*
**Age (years)** (Mean ± SD)	82.9 ± 6.0	80.6 ± 7.4	0.055
**Gender (female)** [*n* (%)]	54 (97.7)	76 (87.4)	0.154
**Family status (married)** [*n* (%)]	12 (21.1)	29 (33.3)	0.114
**Nursing caregiver (yes)** [*n* (%)]	39 (68.4)	48 (55.2)	0.116
*Fracture AO-Type [n (%)]*			
Type A	35 (61.4)	48 (55.2)	0.467
Type B	22 (38.6)	39 (44.8)	
*Medical status*			
CCI TS (Mean ± SD)	5.6 ± 1.9	5.1 ± 1.7	0.081
CCI TCS (Mean ± SD)	1.7 ± 1.7	1.5 ± 1.7	0.319
Medication number (Mean ± SD)	6.7 ± 3.5	6.9 ± 3.8	0.758
MMSE (Mean ± SD)	19.2 ± 5.3	24.2 ± 4.5	<0.0001
Norton (Mean ± SD)	14.0 ± 1.4	14.2 ± 1.3	0.367
BMI (Mean ± SD)	25.2 ± 5.3	24.2 ± 4.0	0.207
*Laboratory*			
Hemoglobin (g/dl) (Mean ± SD)	11.8 ± 1.5	11.6 ± 1.9	0.723
Serum creatinine (mg/dl) (Mean ± SD)	1.0 ± 0.8	1.2 ± 1.0	0.385
Serum urea (mg/dl) (Mean ± SD)	55.4 ± 33.0	54.0 ± 33.6	0.367
Serum albumin (g/dl) (Mean ± SD)	3.6 ± 0.5	3.6 ± 0.5	0.876
*Morbidity during rehabilitation process*			
Depression [*n* (%)]	11 (19.3)	12 (13.8)	0.389
Delirium [*n* (%)]	4 (7.0)	8 (9.2)	0.67
Any infection [*n* (%)]	11 (19.3)	5 (5.7)	0.015
Cardiovascular (IHD, CHF, stroke) [*n* (%)]	4 (7.0)	4 (4.6)	0.555
Thromboembolism [*n* (%)]	0 (0)	1 (1.2)	
Pressure Ulcers [n (%)]	1 (1.8)	2 (2.3)	0.873
**Rehabilitation time (days)** (Mean ± SD)	17.3 ± 1.0	18.9 ± 7.9	0.226

MRFS-R, the Montebello Rehabilitation Factor Score-Revised; CCI TS, the Charlson Co-morbidity Index, total score; CCI TCS, the Charlson Co-morbidity Index, total cumulative score; MMSE, the Mini Mental State Examination; BMI, body mass index; IHD, ischemic heart disease; CHF, congestive heart failure.

A logistic regression model was built to identify independent variables that were significantly associated with successful rehabilitation (Table 4). The model included sex, CCI TS (because its univariate *p* value was below 0.1 (*p* = 0.081) and because it reflects co-morbidity), patient age, and the other variables that reached statistical significance in the univariate analyses. The latter included MMSE and the rate of infection during hospitalization in the Geriatrics Department. In the model (R^2^ = 0.273), only MMSE remained statistically significant at OR = 1.2 (95% CI 1.1–1.3, *p* < 0.0001).

## Discussion

The results of this study show that there are differences between patients who underwent intensive rehabilitation in the Geriatrics Department for PF and those who underwent it for HF. As expected, most of the patients in both groups were women, but the percentage of women was significantly higher in the PF group. The high rate of women with PF is well documented in the literature with a rate higher than 80% in most studies in elderly populations (15, 18–20, 24, 25, 28). In a Swedish study (43) of over 400,000 patients following osteoporotic fractures, the investigators found that the rate of women among PF patients was higher than among HP patients, but that the differences were lower than in the present study.

**Table 4:** Logistic regression analysis predicting MRFS-R ≥ 50 in patients with pelvic fracture.

Variables	OR (95% CI), *P*
Gender (male)	0.59 (0.12–3.0), 0.531
CCI TCS	1.02 (0.80–1.30), 0.863
Any infection	0.50 (0.14–1.87), 0.306
MMSE	1.21 (1.10–1.31), 0.000
Constant	0.004, 0.068

MRFS-R, the Montebello Rehabilitation Factor Score-Revised; CCI TCS, the Charlson Comorbidity Index, total score; MMSE, the Mini Mental State Examination.

In the present study the HF group had a more complex medical history with a higher CCI score, higher rate of medication use, a lower Norton score, and lower kidney function and albumin levels, compared to the PF group. The complication rate was also higher in the HF population. Some differences in the parameters between the PF and HF groups may be attributed to the fact that the HF group was admitted to the Geriatric Department after undergoing surgery and spending several days in the Orthopedic Division, rather than being directly admitted from the Emergency Department, as was the case with the PF patients. This might partially explain the lower kidney function on admission in the HF group, and a higher frequency of complications compared to the PF group. Although the anamnestic pre-fracture functional capacity was similar in the two groups the need for nursing care was higher in the HF patients, a finding that could reflect a higher level of frailty among HF patients. The combination of a high level of frailty and medical complexity in this population could explain the need for a longer rehabilitation period in the HF group to attain a similar functional level. The mortality rate during the first follow-up year was also higher in the HF group. In the study by Lundin et al. (43), the mortality rate over the first post-fracture year in patients over the age of 50 was 25% after HF and 21% after PF. However, another study (12) found that the higher mortality rates among HF patients evened out with PF at 5 years post-fracture. The higher mortality rates over the first year in the HF group could be related to the higher rate of men in this population. Lundin et al. (43) also found that the differences in mortality rates between men and women were higher in the HF group than among PF patients. The burden of illness in the HF group could also explain the difference in mortality. In any event, the mortality rate in the present study over the first post-fracture year among PF patients at 6.3% was lower than in previous studies. In the Swedish study cited above (43), the mortality rate in the PF group was 21% after 1 year. In other studies the mortality rate among patients hospitalized after PF ranged from 9.5 to 27% (15, 18, 23, 28). In a comparison of the PF population in the present study to a previous study of PF patients hospitalized in a Geriatrics Department (20), in addition to the lower mortality rate (6.3% vs. 12.5% in the present study), the infection rate during hospitalization (11.1% vs. 36.7%), and the duration of hospitalization in the department (18.2 vs. 45 days) were also lower in the present population. In a review of the literature we did not find any paper that related specifically to a population of PF patients that were hospitalized for intensive rehabilitation. This may represent a selection bias in the sample of PF patients who were admitted to our department for rehabilitation.

Another aim of the present study was to identify predictors of successful rehabilitation after PF. The present study included 83 patients with Type A PF and 61 with Type B PF. There were no patients with Type C PF. There was no difference between the groups in the outcome of rehabilitation, with 57.8% of patients with Type A PF and 63.9% of patients with Type B PF reaching MRFS≥50. There are several possible explanations for this result. While Type A PFs are stable, stability from the vertical point of view (9) in Type B PF may be sufficient for successful rehabilitation. Of course, the sample size and the retrospective nature of the study could mean that other confounders may be responsible for the lack of difference in rehabilitation between the two groups.

Cognitive state was an independent predictor of rehabilitation success, with every addition of one point in the MMSE score increasing the patient’s chance of reaching MRFS-R ≥ 50 by 20.5%. The association between cognitive state and the outcome of rehabilitation after HF has been reported in many studies in the field (44–49). As noted, no studies assessed the association between cognitive state the outcome of rehabilitation after PF.

In contrast to previous studies of patients who underwent intensive rehabilitation in our department after HF, i.e., with the same staff and identical work routine, factors such as serum albumin level (35) or comorbidity (42) were not found to predict rehabilitation success in PF patients.

The present study has several advantages. It is the first study, to our knowledge, to compare the outcome of intensive rehabilitation among patients with HF and PF, and it is the first attempt to identify independent predictors of rehabilitation outcomes. The study used a single, uniform method to measure rehabilitation success, MRFS-R, which was also used in our earlier studies (35, 42, 45).

## Limitations

The study has limitations. It is a retrospective study that used data from the computerized medical record with a sample of 144 participants. Several variables that might have affected the outcome of rehabilitation including the level of patient motivation and the extent of family participation in the rehabilitation process, were not collected. The follow-up period was only 1 year and there are no data on the patients’ functional levels on discharge from the department or in the long term.

In the present study the sample of the PF groups was compared to the sample of the group of patients with HF that were included in another study (35). Since this group of HF patients was limited to 138, we could not conduct matching between the groups in terms of age, sex, and comorbidity. This circumstance is, without doubt, a study limitation.

Finally, as noted above, due to potential selection bias in admission to the department, it may be difficult to generalize the results of this study to the general population of elderly patients with PF.

In summary, the present study found gaps between patients undergoing rehabilitation after HF and PF, with cognitive state the only predictor of a successful rehabilitation. In light of the increase in the rate of PF in the elderly population there is an urgent need for studies focusing on this condition in that population.

## Data availability statement

The raw data supporting the conclusions of this article will be made available by the authors, without undue reservation.

## Ethics statement

The studies involving humans were approved by Internal Review Board (Helsinki Committee) of the Soroka University Medical Center (Approval # SOR-0466-20; date of registration: 18/05/2021). The studies were conducted in accordance with the local legislation and institutional requirements. Written informed consent for participation was not required from the participants or the participants' legal guardians/next of kin in accordance with the national legislation and institutional requirements.

## Author contributions

DK: Conceptualization, Data curation, Investigation, Writing – original draft. AG: Conceptualization, Data curation, Investigation, Writing – original draft. AA-A: Data curation, Investigation, Writing – original draft. EM: Data curation, Investigation, Writing – original draft. ES: Data curation, Investigation, Writing – original draft. EZ: Conceptualization, Data curation, Investigation, Writing – original draft. DS: Conceptualization, Data curation, Investigation, Writing – original draft. NV: Data curation, Investigation, Writing – original draft. TF: Conceptualization, Data curation, Formal analysis, Methodology, Writing – original draft. YP: Conceptualization, Data curation, Formal analysis, Investigation, Methodology, Project administration, Supervision, Writing – original draft, Writing – review & editing.
